# Role of MHC class I pathways in *Mycobacterium tuberculosis* antigen presentation

**DOI:** 10.3389/fcimb.2023.1107884

**Published:** 2023-03-15

**Authors:** Karolina D. Witt

**Affiliations:** ^1^ Pandemic Sciences Institute, University of Oxford, Oxford, United Kingdom; ^2^ Nuffield Department of Medicine, University of Oxford, Oxford, United Kingdom

**Keywords:** *Mycobacterium tuberculosis*, antigen processing, MHC-I, vaccine, host-pathogen interactions, cytotoxic T cells

## Abstract

MHC class I antigen processing is an underappreciated area of nonviral host–pathogen interactions, bridging both immunology and cell biology, where the pathogen’s natural life cycle involves little presence in the cytoplasm. The effective response to MHC-I foreign antigen presentation is not only cell death but also phenotypic changes in other cells and stimulation of the memory cells ready for the next antigen reoccurrence. This review looks at the MHC-I antigen processing pathway and potential alternative sources of the antigens, focusing on *Mycobacterium tuberculosis* (*Mtb*) as an intracellular pathogen that co-evolved with humans and developed an array of decoy strategies to survive in a hostile environment by manipulating host immunity to its own advantage. As that happens *via* the selective antigen presentation process, reinforcement of the effective antigen recognition on MHC-I molecules may stimulate subsets of effector cells that act earlier and more locally. Vaccines against tuberculosis (TB) could potentially eliminate this disease, yet their development has been slow, and success is limited in the context of this global disease’s spread. This review’s conclusions set out potential directions for MHC-I-focused approaches for the next generation of vaccines.

## Introduction

*Mycobacterium tuberculosis* (*Mtb*) is a causative agent of tuberculosis—an infectious disease responsible for ten million cases and over a million deaths every year (WHO, 2020). Despite the availability of antibiotics (Paulson, 2013) and vaccination with an attenuated form of *Mycobacterium bovis*, the Bacilli Calmette-Guérin (BCG), TB eventually kills 45% of HIV-negative people and nearly all HIV-positive individuals (WHO Key Facts, 2022).

Major histocompatibility complex receptor classes I and II (MHC-I and MHC-II) are two families of receptors involved in the recognition of “self” and surveillance of “foreign” antigens. While class II receptors evolved for the defense against pathogens and are present primarily in the immune cells, class I receptors are much more ubiquitous, also on MHC-II-negative cells. Ubiquitous presence may play a unique role in multiple-tissue surveillance, pathogen detection, and restriction of disease dissemination. MHC-I (or HLA class I) contributes both to innate immunity through the engagement of natural killer (NK) cells and to adaptive immunity through peptides presented to cytotoxic T cells (Jiang et al., 2019; Uzhachenko and Shanker, 2019). In contrast, MHC-II is solely dedicated to adaptive immunity (Leddon and Sant, 2010).

MHC-I molecules constitute a fundamental aspect of self in self-organizing networks of multicellular organisms starting from jawed vertebrates. Self is a process of continuous rebalancing of different biochemical and intercellular interactions rather than a constitutive feature of a single cell, as proposed initially by Turing (1990). MHC-I molecules present a snapshot of the intracellular proteome from all cell compartments by uploading self-peptides, byproducts of the reactions taking place in the cytosol. This proteome is recognized by T cells selected both in the thymus and periphery to have autoreactive cells eliminated or repressed (Perreault, 2010). MHC-I molecules can also present self-peptides that are mutated, as well as pathogen-derived mimicry peptides (Trost et al., 2010). The aim of the antigen presentation is the recurring recognition of cumulative signals up to the threshold where the “non-self” cell is destructed by adaptive immune cells, mainly CD8^+^ T cells, while protecting healthy cells from NK-mediated cytolysis. This requires coordinated action involving a series of brief encounters between antigen-presenting cells and their immune counterparts. As MHC-I molecules feature on every nucleated cell of the organism, they visualize pathological processes that occur in the organism at an early stage. Peptide presentation on MHC-I molecules, recognition by T-cell receptors (TCR), and immune cell activation are three stages in which cell interactions are inspected for affinity binding and initial signal strength, duration of stimulation, and decay (Segura et al., 2008; Pathni et al., 2022). Historically, the MHC-I system has been linked to transplant immunology and viral infections. MHC-I antigen presentation may contribute to the development of new vaccination strategies against chronic bacterial infections.

## Structure and diversity of MHC-I molecules in macrophage

The major histocompatibility complex (MHC) locus, found on the short arm of chromosome 6 in humans, encodes genes from three classes of proteins (MHC-I, MHC-II, and MHC-III). There are three MHC-I groups, termed classical human leukocyte antigens A, B, and C (HLA), and several non-classical HLA groups, such as HLA-E, F, and G, a cluster of differentiation 1 (CD1) molecules, and MHC-I-related protein (MR1). Classical MHC-I molecules show a high degree of polymorphism, resulting in allele diversity predicted to encompass eight to nine million variants, although 80% of these occur only rarely and are represented by alleles differing by single base point mutations (Robinson et al., 2017). In humans, the most commonly found alleles are clustered into three major groups A–C, and 42 core classical types further grouped into 12 supertypes based on the similarities in peptide-binding motifs (Sette and Sidney, 1999). HLA class I supertypes have been linked to susceptibility and severity of tuberculosis (Balamurugan et al., 2004). Conversely, genes coding nonclassical HLA-E, HLA-F, and HLA-G are highly conserved as one or two alleles, often tissue-restricted, and involved in molecular mechanisms underpinning immune tolerance and fetus acceptance in pregnancy (Moscoso et al., 2006). Tissue expression of MHC-I molecules varies. In some cell subsets, such as postmitotic neurons, it is very limited (Neumann et al., 1997); in others, such as lymphocytes, MHC-I constitutive expression reaches up to 1% of the membrane protein content (Joly et al., 1991). It is also subject to transcriptional activation regulators TAF1, USF1/2, and CIITA (Howcroft et al., 2003).

Classical MHC-I molecules are made with a heavy glycoprotein α chain, composed of three immunoglobulin-like domains α-1, α-2, and α-3, a transmembrane segment with a cytosolic tail, and a smaller noncovalently attached light β chain (β2-microglobulin). Processed antigen fragments in the form of 8 to 10 amino acid peptide chains are affinity bound with each amino acid contributing to the overall MHC affinity score (Lundegaard et al., 2010). The binding pocket is formed by two conformational domains of the heavy chain, α1 and α2, supported by the β2 chain and its own residues to prevent binding of the longer peptides that would need to bulge outside the pocket. There are usually two anchor residues within the octo- or nonameric peptide, one of which is predominantly at the C-terminus (Falk and Rötzschke, 1993). It is different in MHC-II class molecules where two similarly sized α and β chains form an open-ended binding site so that typically 13–18 amino acid long peptides can extend out (Germain, 1994). In both MHC-I and MHC-II molecules, there are pockets present in the binding groove that accommodate side chains of the peptides; physicochemical features of these pockets underpin allele-specific consensus motifs (Falk et al., 1991). In MHC-I molecules, the conformational stability of the whole molecule is equally dependent on MHC and the bound peptide; it disassociates with any of the components’ removal, leading to MHC-I recycling from the cell surface. The peptide-MHC (pMHC) complex is recognized by the T-cell receptors and its CD8^+^ co-receptor, which binds to the nonpolymorphic α3 domain in the heavy chain (Huppa et al., 2010).

Nonclassical MHC-like, MHC-Ib, or MHC-related molecules resemble the classical receptors with their heavy chains but do not always associate with β2 microglobulin, forming homodimers instead. They include MR1, HLA-E, HLA-F, HLA-G, and CD1 molecules. MR1 is an MHC-Ib molecule that uniquely binds small molecules of the microbial metabolome. Several isoforms of MR1 have been detected, albeit only one, MR1A bears a close resemblance to the classical MHC-I molecules and is fully functional (Riegert et al., 1998). Other isoforms are either nonfunctional or with uncertain properties, like MR1B, which forms homodimers that do not associate with the β2 light chain. Although these isoforms may not be functional in the antigen-presenting process, they can still play role in intracellular trafficking. HLA-E or Qa-1 binds leader sequences of other MHC-I molecules and, as such, is a sensor for their expression and checkpoint in antigen presentation to NK cells. In homeostasis, HLA-E binds only a restricted set of nonamers (O’Callaghan and Bell, 1998).

The CD1 proteins’ binding groove is deeper and lined almost entirely with nonpolar or hydrophobic amino acid chains. This groove can accommodate a long hydrophobic lipid tail inside its pockets and expose the hydrophilic part on the surface, where it directly contacts the T-cell antigen receptor. In contrast to MHC-I molecules, where the peptides are trimmed to 9–11Aa-long peptides, lipids bound by CD1 proteins are not cleaved but adapted within the hydrophobic clefts, which can accommodate up to C70–C80-long chains. In humans, there are four members of antigen-presenting CD proteins: CD1a, CD1b, CD1c, and CD1d, and CD1e, which is a soluble carrier in the endolysosomal network for other CD1-lipid complexes (Moody et al., 1999; Ly and Moody, 2014; Moody and Suliman, 2017).

Finally, for completeness, in humans, there are MHC class I chain-related molecules that are not involved in antigen presentation but are still involved in immune responses. These are MICA and EPCR proteins, with MICA being highly polymorphic glycoproteins that are expressed ubiquitously and serve as a “danger signal” for NK cells, γδ T cells, and CD8^+^ T cells *via* NKG2D receptors (Schrambach et al., 2007). EPCR, a 46-kDa protein, shares ~20% homology with the CD1d molecule. They are expressed in the vascular system (endothelium) as well as on various innate immune cells; they are ligands for TCR of Vδ2 γδ T cells. MICA and EPCR proteins, respectively, regulate inflammation and coagulation (Willcox et al., 2012).

## Mycobacterium tuberculosis as an intracellular microbe: Role of the phagosome

**Figure 1:**
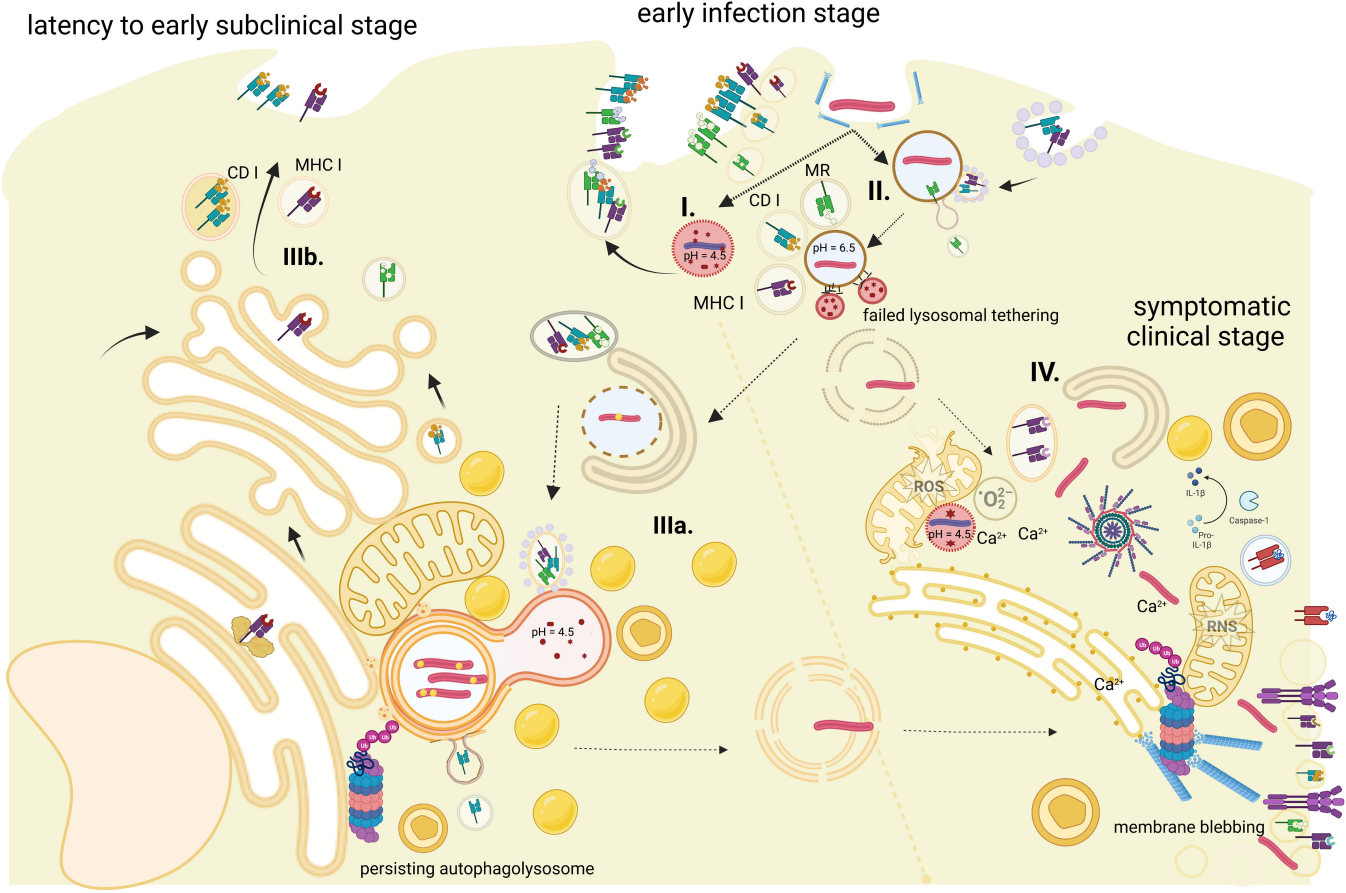
Antigen processing for MHC-I presentation during the course of *Mtb* infection. The figure represents stages of phagocytosed bacilli (pathways are time- and site-specific). (I) The phagolysosome, which disables pathogen—presented antigens are from fragmented dead bacilli. (II) Persistent phagosomes where fusion with lysosome was blocked successfully by the bacilli—presented antigens are virulent factors released by live *Mtb*. (III) Autophagolysosomes with persistent latent bacilli—presented antigens are scanty and a by-product of pathogen–host cellular organelles interactions: (IIIa) stage of autophagosome nucleation initiation and (IIIb) a stage of persisting autophagolysosome *via* contact sites with ER and classical MHC-I antigen processing route. (IV) Cytosol bacilli—stage of active infection with *Mtb* overtaking cell innate defense, generalized disruption of cell functions, and likely progress to cell death. Dashed arrows represent pathogen transition; solid arrows represent sources of the antigen. Created with BioRender.com.

*Mtb* is an intracellular microbe that can infect any tissue, but the lung is the main niche for its transmission (Torrelles and Schlesinger, 2017). The presence of the asymptomatic latent phase in a significant proportion of immunocompetent infected individuals makes this pathogen life cycle akin to other human pathobionts that cause harm only under host–pathogen disequilibrium (Hakansson et al., 2018). The typical *Mtb* life cycle includes a phase of the primary disease followed by a stage of long-term occult and persistent intracellular infection. During this “latent” period, bacilli survive in a non-vegetative dormant form characterized by a thick lipid-rich bacterial cell wall and lipid accumulations (Vázquez et al., 2014) protecting from the degrading activity of host autophagolysosomal enzymes (**Figure 1**).

Alveolar macrophages serve as the first port of entry and primary host for *Mtb*, killing it and presenting antigens while also sheltering persistent bacilli (van Crevel et al., 2003). The process of phagosomal uptake involves a range of cell receptors, including cell surface pattern recognition receptors (PRR), which bind pathogen-associated molecular patterns (PAMPs). Toll-like receptors (TLRs)on the cell surface (TLR1, TLR2, TLR4, TLR6) and endosomal membranes (TLR3, TLR7, TLR8, TLR9) are key PRRs that are responsible for the induction of the signaling pathways downstream and the production of cytokines (Underhill et al., 1999). Interestingly, mycobacteria-led use of TLR3 appears to enhance IL-10 production (Bai et al., 2014), suggesting that the binding of *Mtb* RNA may influence cross-talk with other intracellular signaling pathways. It has also been shown that other receptors, like C-type lectin receptors, scavenger receptors, nucleotide-binding-oligomerization domain (NOD)-like receptors (NLRs), opsonin receptors, ficolin, C1q, complement receptors CR1, CR3, and CR4, and Fc receptors for bacilli opsonized with immunoglobulins, promote phagocytosis but not necessarily pathogen eradication (Hossain and Norazmi, 2013).

The arrest of phagosome maturation is an important stage in the *Mtb* life cycle (**Figure 1**). *Mycobacterium tuberculosis* influences acidification of the phagosome in the early phase of infection by preventing tethering of V-ATPase to *Mtb*-containing vacuole and stabilizing its pH at 6.3–6.5 with tyrosine phosphatase PtpA (Wong et al., 2011). Early phagosomes undergo a series of encounters with other endocytic organelles to acquire various molecules, Rab5, EEA1, PI(3)P, and VPS 34 to name a few, before they progress to the late phagosome stage. *Mtb* prolongs that stage by hydrolyzing PI3P by the action of PI3P-specific acid phosphatase SapM (Vergne et al., 2005); mycobacterial ManLAM blocks the transport of acidic cargo from the trans-Golgi network *via* interference with early endosomal antigen EEA1-syntaxin 6 interactions (Fratti et al., 2003) and protein kinase G (pknG) induces continued accumulation of Rab5 and prevents Rab7 acquisition, further delaying phagosome maturation (Roberts et al., 2006). That temporary blocking effect is eventually overcome by the IFN-γ-activated macrophage. The immune response to mycobacterial early-conserved immunodominant epitopes of the *Esx* secretion system triggers pH to drop to 5.0 in the phagolysosome. However, the time gained allows *Mtb* to alter its ability to use carbon sources with the preference of glucose usage and storage of neutral lipids (triacylglycerols (TAG)) in droplets (Vázquez et al., 2014) followed by mycobacterial entry into the dormant phase.

### Early phagosomal stage

During the early stage, phagosomes fuse with recycling endosomes containing classical MHC-I molecules endocytosed from the cell surface and traffic nonclassical MR1 molecules from the *trans*-Golgi network (Harriff et al., 2016). *Mtb* is thought to directly load its antigens on these MHC-I molecules and release them to the cell surface without going through the proteasomal and cytosolic processing route. Peptides in the phagosomes are derived from the action of phagosomal proteases, in particular, cathepsins belonging to aspartic (D, E), cysteine (B, C, F, H, K, L, O, S, V, X, and W), and serine (A and G) proteases (Pires et al., 2016). *Mycobacterium tuberculosis* was shown to downregulate not only cathepsins but also cathepsin inhibitors and cystatins. In effect, the antigen processing activity may actually be improved, as it was shown that the high proteolytic potential of these enzymes leads otherwise to epitope destruction *via* cleavage into extremely short peptide sequences no longer able to anchor into the binding groove of MHC-I molecules (Moss et al., 2005). The loading process is enhanced by endosomal TLR signaling and supported by a number of accessory molecules, some stationary for phagosome and others shuttled from the endoplasmic reticulum (ER). There is little knowledge about the “quality” of presented antigens in respect of infected cell recognition and destruction; however, the tight control over the timing of lysosome fusion by the pathogen may favor antigen processing of small, highly immunodominant proteins from the *Esx* family with, possibly, a role for some members of proline-glutamate (PE) motif-containing PE-PPE family of mycobacterial proteins (Vordermeier et al., 2012) in the absence of key lysosomal proteases—some of these virulence factors are listed in **Table 1**.

**Table 1:** Selected *Mycobacterium tuberculosis* virulence factors participating in host virulence resistance.

Protein	Size	Role	Putative location	Reference
Phagosome immunodominance				
SapM (Rv3310)	299aa	Dephosphorylates phosphatidylinositol 3-phosphate (PI3P) catalyzing its hydrolysis; inhibition of phagosome maturation; binding to the small GTPase RAB7 delaying autophagy flux	Phagosome	Vergne et al. (2005) and Hu et al. (2015)
PPE57 (Rv3425)	176aa	TLR2 stimulation/proinflammatory cytokines/macrophage maturation with upregulation of MHC-II	Phagosome	Xu et al. (2015)
PPE17 (Rv1168)	346aa	TLR2 stimulation/proinflammatory cytokines; immunodominant epitopes	Phagosome	Udgata et al. (2016)
PPE39 (Rv2353)	354aa	Enhances macrophage maturation and upregulation of MHC-I and MHC-II molecules; induces production of proinflammatory cytokines and Th1 responses	Phagosome	Choi et al. (2019)
PE-PGRS11 (Rv0754)	584aa	TLR2, proinflammatory cytokines and Cox2 expression stimulation; induction of antiapoptotic Bcl2	Phagosome, autophagosome	Bansal et al. (2010) and Chaturvedi et al. (2010)
Ppa (Rv3628)	162aa	TLR2 stimulation/proinflammatory cytokines and Th1 immune responses	Phagosome	Kim et al. (2016)
Rv1507A (Rv1507)	231aa	Induction of proinflammatory cytokines and upregulation of MHC-I and MHC-II molecules	Phagosome	Arora et al. (2020)
TB27.3/cfp32 (Rv0577)	261aa	TLR2 stimulation/proinflammatory cytokines/upregulation of MHC-I and MHC-II on DCs	Phagosome, cell exit	Byun et al. (2012)
EsxL (Rv1198)	94aa	TLR2 stimulation/TNF-α, IL-6 production	Phagosome	Pattanaik et al. (2021)
EspC (Rv3615)	103aa	Stimulation of proinflammatory cytokines; contains broadly recognized CD4^+^ and CD8^+^T-cell epitopes	Phagosome, cell exit	Millington et al. (2011)
EsxV-EsxW (Rv3619/20)	94aa-98aa	Elicits increased levels of IFN-gamma, IL-12, and IgG(2a) as a dimer	Phagosome, cell exit	Mahmood et al. (2011)
Persistence				
DnaK (Rv0350)		Bacterial chaperone protein stimulates macrophage for higher arginase activity, diverts it from the iNOS pathway, and switches on IL-10 production	Autophagosome	Lopes et al. (2016)
PtpA (Rv2234)	163aa	Dephosphorylates host VPS33B protein, which induces a block of the host phagosome maturation; antagonizes host protein TRIM27, which acts as E3 ubiquitin ligase that promotes innate immune responses and cell apoptosis	Phagosome, Autophagosome	Wang et al. (2016)
CpsY-cpsA (Rv0806/Rv3484)	532aa	Glucose epimerases and stealth proteins conserved from bacteria to higher eukaryotes; diverting host glycosylation pathways; CpsA prevents recruitment of NADPH oxidase to the phagosome	Autophagosome	Sperisen et al. (2005) and Köster et al. (2017)
PPE34 (Rv1917)	1459aa	Induced maturation of dendritic cells, *via* antigen presentation, induces Th2 responses with IL-10 production	Autophagosome	Bansal et al. (2010)
Erm37 (Rv1988)	179aa	Localized to host chromatin serving as a functional methyltransferase that demethylates an arginine residue at H3R42 to repress a range of host genes involved in reactive oxygen species (ROS)	Autophagosome	Yaseen et al. (2015)
Eis (Rv4216)	402aa	Secreted protein, which increases acetylation of host histone H3 to upregulate IL-10 and suppress autophagy and inhibits ERK1/2, JAK pathway, and subsequent production of tumor necrosis factor-alpha (TNF-alpha) and interleukin-4 (IL-4); inhibits ROS production *via* acetylation of host DUSP/MKP-7 phosphatase	Autophagosome	Lella and Sharma (2007); Samuel et al. (2007), and Kim et al. (2012)
LprG (Rv1411)	236aa	Lipoprotein inhibits MHC-II antigen processing, enhances recognition of Mtb acetylated glycolipids by TLR2, induces mitochondrial fission, and lowers respiratory cell rate	Autophagosome	Gehring et al. (2004); Drage et al. (2010), and Aguilar-López et al. (2019)
PPE18 (Rv1196)	391aa	Downregulation of Th1 responses, upregulation of Th2 responses	Autophagosome	Nair et al. (2009); Bhat et al. (2012), and Udgata et al. (2016)
PE-PGRS30 (Rv1651)	1011aa	Downregulation of Th1 responses, phagolysosome block	Phagosome, Autophagosome	Chatrath et al. (2016)
PPE2 (Rv0256)	556aa	Contains a eukaryotic-like nuclear signal, which allows it to be directly translocated into the host nucleus, where it binds to the NOS2 promoter and limits host ROS production	Autophagosome	Bhat et al. (2017)
TlyA (Rv1694)	268aa	Downregulation of Th1 and Th17 responses	Autophagosome	Rahman et al. (2015)
LpqT (Rv1016)	226aa	Mannosylated protein; downregulation of Th1 and Th17 responses; inhibits maturation of dendritic cells and decreases the production of proinflammatory cytokines	Autophagosome	Su et al. (2019)
LpnQ (Rv0583)	228aa	Secreted, directly interacts with the human E3 ubiquitin ligase CBL	Autophagosome	Penn et al. (2018)
PE35/PPE68 (Rv3872/73)	99aa/368aa	Located in RD1 region; stimulates the secretion of IL-10 and MCP-1 *via* TLR2 activation	Autophagosome	Tiwari et al. (2014)
DlaT (Rv2215)	553aa	Function with Lpd as NADH-dependent peroxidase and peroxynitrite reductase that provides protection against nitrosative stress	Phagosome, autophagolysosome	Shi and Ehrt (2006)
Mce2D (Rv0592)	508aa	Less TNF-α and IL-6; all mce1–4 take part in adaptation to adverse conditions of autophagosome	Autophagosome	Singh et al. (2016)
Cell exit				
PE-PGRS33 (Rv1818)	236aa/498aa	Influence mitochondrial dynamics; precipitates macrophage apoptosis *via* mitochondrial CytC activation, leading to an increase in caspase-3 and caspase-9, induces TNF-α and TNF receptors 1A; highly immunodominant for both cellular and humoral responses	Cell exit	Basu et al. (2007); Cohen et al. (2014), and Aguilar-López et al. (2019)
LpqH (Rv3763)	159aa	Induces interleukin 1-beta and IL-12 p40 (IL12B) and TNF-α; inhibits MHC-II expression and antigen processing in the host; traffics *via* MHC-I processing pathway and *via* bacilli extracytoplasmic vesicles; may reduce vacuolar MHC-I processing; induces macrophage apoptosis *via* loss of mitochondrial membrane potential, release cytochrome *c*, and release of mitochondrial apoptosis-inducing factor AIF followed by upregulation of death receptor signaling and caspase-8 and caspase-3	Autophagosome, cell exit	Noss et al. (2001); Tobian et al. (2003); Stewart et al. (2005), and Sánchez et al. (2012)
PE-PGRS17 (Rv0978)	331aa	TLR2 stimulation/proinflammatory cytokines	Phagosome, cell exit	Chen et al. (2013) and Moodley et al. (2022)
EsxT (Rv3444)	100a	Induces macrophage apoptosis *via* activation of NF-kappa-B and tumor necrosis factor-related apoptosis-inducing ligand (TRAIL); secreted *via* esx-4 T7S	Cell exit	Shi et al. (2014)
EspB (Rv3881)	460aa	The secreted processed form of EspB binds to phosphatidic acid and phosphatidylserine-inducing host death; inhibits IFN-gamma-induced autophagy; binds to human serum amyloid A, acute phase protein; and facilitates cell entry of opsonized Mtb	Cell exit	Chen et al. (2013); Huang and Bao (2016); Kawka et al. (2021)
PE9-PE10 (Rv1088/89)	144/120aa	Induces macrophage apoptosis, downregulation of IL-1B increases IFNB, has a number of highly immunodominant T-cell epitopes	Cell exit	Tiwari et al. (2015) and Sunita et al. (2020)
PE25/PPE41 (Rv2430/31)	194aa/99aa	As dimer induces macrophage necrosis, induces maturation of mouse dendritic cells, and drives Th2-biased immune responses	Cell exit	Tundup et al. (2014) and Chen et al. (2016)

### Late phagosomal stage

During the late phagolysosome stage, more lipids are present in the phagolysosome, which is reflected in antigen presentation shifting from proteins to lipids. It was shown that CD1d and CD1b molecules have an endosome-targeting motif regulated also by the interaction with MHC-II complexes and are able to withstand lower pH in the phagolysosome (Jayawardena-Wolf et al., 2001). Antigenic lipid-CD1 complexes traffic to the cell surface for iNKT cell activation (Sillé et al., 2011).

### Cytosolic bacteria

Bacilli that escape from phagosomes or “leaky” phagosomes do so through the damaged primary phagosome membrane, using virulence factors and activating host cytoplasmic phospholipase A2 (cPLA2). Activation of cPLA2 has the additional effect of releasing arachidonic acid from plasma membranes. Arachidonic acid is the precursor of small lipid molecules and eicosanoid biosynthetic pathway derivates: lipoxins, prostaglandins, and leukotrienes. Their mechanisms of action create a network of regulatory counterbalances. Prostaglandins (PGE4) promote plasma membrane repair, whereas lipoxin A4 supports mitochondrial damage and macrophage necrosis *via* inhibition of PGE4-producing cyclooxygenase 2 (Chen et al., 2008). Virulent strains of *Mtb* have more propensity to inhibit macrophage apoptosis and antigen cross-presentation stimulating 5-lipoxygenase-dependent pathways (Divangahi et al., 2010; Jamwal et al., 2016). The burden of escaping cytosolic bacilli depends on their intra-phagosomal replication, which is higher for more virulent strains and lower for effective innate responses that either induce *Mtb* dormancy or lead to bacilli killing. Many cytosolic bacilli and partially damaged phagolysosomes are captured by a system of intracellular membranes which restructures into double-membrane autophagosomes. It is characterized by a series of sequential steps: nucleation, elongation, and completion. ER, mitochondria, and other organelles serve as sources of membranes and various other molecules facilitating the transfer of MHC-II proteins into the intravesical lumen (ten Broeke et al., 2013; Kimmey and Stallings, 2016).

### Persistence of the autophagosome

The inhospitable environment of the autophagosome and autophagolysosome leads bacilli to re-enter their dormant form upon activation of the two-component transcriptional program dormancy-survival regulator, DosR-DosS, by hypoxic conditions. The acidic environment of autophagolysosomes contains enzymes that cause osmotic and redox damage. Bacilli become resistant, building up intracellular lipid content, thickening cell capsule, metabolic downshift, and glyoxylate shunt for efficient maintenance of tricarboxylic cycle (TCA) components and low levels of DNA synthesis (Lipworth et al., 2016; Murima et al., 2016). Eventually, persistent bacilli are thought to become nonreplicating, metabolically inert, and survive as hidden from host subpopulations. Suppressed metabolism and bacilli dormancy decrease overall antigen presentation to the host and impair host lytic attack to disassemble bacilli. While still confined, the secreted pathogen antigens that get through the autophagosome membrane are thought to have molecular signal sequences that direct them into different intracellular compartments where they influence host metabolism and respiration (Jamwal et al., 2013). These molecules, often PE/PPE proteins, are smaller in quantity but have structural modifications that disable efficient cytosol processing of MHC-I molecules (Koh et al., 2009; Saini et al., 2016). Some of these molecules are listed in **Table 1**. While it was reported that bacilli resuscitation may happen a number of years after the initial infection, the majority of current reports indicate a period of about 2 years (Behr et al., 2018). Beyond that, the control of dormant bacilli over the host macrophage metabolism, if not actively progressed, fades away, resulting in infection resolution (Stephenson and Byard, 2020).

### Cell exit

Periodically, or upon a trigger such as a drop in immune surveillance, resuscitating and then actively replicating bacilli puncture the autophagosome membranes with its pore-forming virulence factors from the *esx* family. Bacilli release membrane vesicles (Prados-Rosales et al., 2011) that contain a high density of acetylated PIM, phospholipids, polyacetylated trehalose, and phenolic glycolipids (PGL), as well as *Mtb* virulence factors—Ag85 complex, CFP10, and lipoproteins (LPR family, PstS1). Microdisruptions of the cell membrane elicit immediate cell response to re-seal the lesion, maintain the continuity of the membrane, or form a new autophagosome to prevent the acidic content from leaking out, risking cell death (Zhen et al., 2021). Host proteins involved in either sensing membrane damage or its repair processes, like synaptotagmin Syt7, galectins Gal 3, Gal9, Annexin, SNARE proteins, and VPS4 are also known to be selectively disrupted by *Mycobacterium tuberculosis* (Gan et al., 2008; Divangahi et al., 2009; Chávez-Galán et al., 2017; López-Jiménez et al., 2018). Subsequently, infected macrophages may undergo a process of remodeling and fusion into Langhans multinucleate giant cells, with the possible contribution of phenolic glycolipids on the bacilli part (Cambier et al., 2017). The process of disruption of intracellular membrane continuity may also lead to cell death *via* either apoptosis, necrosis (Divangahi et al., 2009), pyroptosis (Behar et al., 2010), or ferroptosis (Amaral et al., 2019)—each endowed with separate characteristics that determine the degree of antigen cross-presentation uptake from host membrane exosomes (Giri et al., 2010) or engulfment by other phagocytes (Gan et al., 2008; Amaral et al., 2019). The possible routes of host–pathogen interactions are schematically represented in **Figure 1**. Bacilli persistence is a granuloma-specific phenomenon, as is bacilli resuscitation and return to active replication upon a change in the environmental conditions. Bacilli resuscitation in small quantities may be also a stochastic process (Buerger et al., 2012). While the former is a more generalized event and a result of weakening immune responses (Riaño et al., 2012), the latter might be more localized, confined to several bacilli that show differential patterns of resuscitation factors (Mukamolova et al., 2002; Tantivitayakul et al., 2020) and DosR-S regulon expression (Domenech et al., 2017). Successful “scouts” active at the border between the necrotic center and host cellular wall of defense (Davies et al., 2008) spread out to set up distant tubercle satellites in other lung lobes or cause multiple foci of inflammation in miliary tuberculosis (Liu et al., 2015).

## Beyond phagosome: Sources of the antigen for MHC-I presentation in *Mtb* infection

1. Autophagosome-to-cytosol pathway: small molecules. Various molecules are actively secreted by live *Mtb* cells. Nearly 30% of the *Mtb* proteome is composed of small proteins defined as <200Aa. Development of the strategies that disable these molecules facilitates processing and antigen presentation. If the autophagosome establishes direct membrane contact with ER *via* fusion (Guermonprez et al., 2003), smaller size molecules (**Table 1**) can be transported out of the autophagosome either *via* ER-specific sec61 channel into ER, where post-translational modification are removed and then out to cytosol (Römisch, 2017). Retrograde transit facilitates entry of these molecules to the 19S unit of the proteasome and then back to the ER to form pMHC-I complexes *via* an antigen-processing transporter (TAP)-dependent pathway. Candidates could include *Mtb* 19-kDa glycosylated lipoprotein LpqH, fibronectin attachment protein Mpt32, and superoxide dismutase SodC (Mehaffy et al., 2019). Specific Mtb proteins lack fixed conformational structure, e.g., the PGRS part in PE-PGRS proteins or the PPE part in PE-PPE proteins. This results in their nonspecific interference with host signaling pathways by allosteric mimicry (Sharma et al., 2022). Cytosolic polyubiquitination of lysine residues in hydrophobic stretches of these proteins stabilizes their structure while directing them to the proteasome. Finally, the mycobacterial counterpart to the host sec61 channel is 8 membrane-bound Sec proteins that form translocation apparatus transporting many of Mtb-secreted proteins. In conjunctions with pore-forming type VII secretion system of esx1, esx3, and esx5-sec transport circumvent host ER sec-61 by directing small proteins out of *Mtb* and *via* punctured membrane into the cytosol (Divangahi et al., 2009).

2. Autophagosome-to-cytosol pathway: bigger molecules. Partially digested bacterial cells, e.g., BCG, molecular complexes, or even whole live bacilli, are transferred from the autophagosome to the cytosol in any conditions that significantly impair phagosome membrane stability, as described previously in *Mycobacterium tuberculosis* as an intracellular microbe: role of the phagosome. It was previously shown that in BCG-infected phagosomes, molecules as large as 70 kDa could access the cytosol (Teitelbaum et al., 1999), which may happen at a late stage when the phagolysosome membrane is partially degraded. Of note, both sec61 active transport and membrane disruption mode are not mutually exclusive. The repair patches of the plasma membrane are of ER origin and therefore sources of embedded molecules that may not be characteristic of the endosomal membrane itself.

3. Endosomal processing pathways. Mtb persistence in the phagosome (*Mycobacterium tuberculosis* as an intracellular microbe: role of the phagosome) is linked to the inhibition of its acidification. Less harsh conditions increase the likelihood of early antigens loading on classical MHC-I molecules. Conversely, loading on CD1 requires displacement of smaller self-lipids by bigger Mtb lipids in low pH, characteristic of late phagolysosomes (Ly and Moody, 2014), and is supported by CD1d and CD1b. Peptide-MHC-I loading is enhanced by endosomal TLR3, TLR7, an TLR9 signaling and any additional transport of surface recycled MHC-I vesicles or ER vesicles that may fuse with phagosomes/phagolysosomes and provide clusters of MHC-I pathway components. One of those is the TAP supplied in retrograde transport between endosome and ER (Harriff et al., 2013). In fact, in certain cases, ER vesicles with peptides processed in the cytosol can fuse to phagosomes for further antigen processing and MHC-I loading (Guermonprez et al., 2003; Gardiner et al., 2013). Late endosomes were also reported to be involved in MR1-specific molecule trafficking and release to the cell surface (Harriff et al., 2014).

Uptake of the antigenic molecules from apoptotic (apoptotic blebs, exosomes) or necrotic infected cells by dendritic cells, specifically CD8^+^, takes place *via* phagocytosis, receptor-mediated endocytosis, and micropinocytosis. Exosomes contain various cell wall and membrane byproducts, i.e., PIM, LAM, LM, lipoproteins (LpqH), trehalose dimycolate, and monoglycosylated PGL (Layre, 2020). Dendritic cells have an extensive network of specialized vesicular transport pathways (Montealegre and Van Endert, 2019). While this route is a critical source of *Mtb* pMHC-I complex presentation to CD8^+^ cells, it is less influenced by live intracellular bacilli, which disrupt phagosomal antigen processing and interfere with antigens present in the cytosol (Cruz et al., 2017).

## Immune surveillance of mycobacterial antigen presentation on MHC-I molecules

*Mtb* survival strategy as an intracellular pathogen deploys various, often synergizing mechanisms to manipulate antigen presentation. Part of decoy strategies forms a tactic to “be recognized but not eradicated,” counteracting its host drive to “recognize and eliminate.” Cellular immunity is primarily involved in the local control of the infection *via* a cytokine-mediated feedback loop between antigen-presenting cells (APCs) and T cells. Sentinel lymph nodes, defined as first granuloma-draining lymph nodes (Nieweg et al., 2001), are the site for priming and expansion of the cognate T cells; they are also the main residence of various unconventional subsets of T cells that act as tissue-resident immunity. Lymph nodes can harbor pathogens themselves, transported here from lung parenchyma and directly infecting lymphatic endothelial cells (Lerner et al., 2016).

Cytotoxic T cells are the main responders to MHC-I presentation. The majority of them are classically (MHC-Ia) restricted; these are CD8^+^ T cells that recognize pMHC-I complex *via* TCR. Upon activation, they produce cytotoxic granules and discharge their content *via* direct contact with the target cell; they can also produce Th1-type cytokines IFN-γ, TNF-α, and IL-2. The functional profile of cytokine secretion differs between active and latent TB varying from poly- to monofunctional T cells (Rozot et al., 2013). CD8^+^ T cells preferentially recognize and destroy heavily infected macrophages and represent the sensor of the intracellular bacilli burden (Lewinsohn et al., 2003); or conversely, only heavily infected macrophages are able to stimulate cytotoxic T-cell granule exocytosis. Uniquely, CD8^+^ T cells recognize pMHC-I complexes on MHC-II-negative infected cells like some epithelia and can limit infection propagation in the local alveolar environment (Harriff et al., 2014), at least at an early stage. It is known that *Mtb*-infected cells, albeit recognized, do not elicit effective cytotoxic responses until late in infection, which hints at a possible decoy strategy of the bacilli, known to have many conserved T-cell epitopes (Comas et al., 2010). This appears to be the case for the TB10.4 (EsxH) antigen. It elicits a dominant CD8^+^ T-cell response which poorly recognizes *Mtb*-infected macrophages and is unable to lyse them (Yang et al., 2018). A substantial proportion of TB10.4-specific CD8^+^ T cells are directed to a single epitope, TB10.4_4-11_, at the start of this 96aa small protein sequence, overshadowing other epitopes. This is an interesting example of a diversion strategy against CD8^+^ T cells. As intact TB10.4 or EsxH is reported to inhibit the endosomal sorting complex required for transport (ESCR) that processes antigens for MHC-II epitopes loading in phagosomes (Portal-Celhay et al., 2016), the gain is doubled.

A smaller population of unconventional cytotoxic T cells is MHC-Ib (HLA-E-H, CD1, MR1) restricted. HLA-E presents peptide sequences from other MHC-I molecules, i.e., VMAPRTLIL, VMAPRTLVL, VMAPRTLLL, VMAPRALLL, and VMAPRTLTL (O’Callaghan et al., 1998); it was reported that HLA-E may present a set of *Mtb*-derived peptides, i.e., VMATRRNVL, VLRPGGHFL, VMTTVLATL, and RLPAKAPLL (Caccamo et al., 2015), some with striking similarities to self-derived leader sequences. Among HLA-E-restricted nonclassical tolerogenic T cells, there has been also a smaller subset identified with Th2 cytokines (IL-4,5,13) secreting properties that activate B cells (Joosten et al., 2010). These cells, upon recognizing Mtb epitopes presented on HLA-E, are not only diverted from their cytolytic functions but induced secretion of cytokines is also acting to suppress other T cells in the vicinity (van Meijgaarden et al., 2015).

CD1 molecules are highly conserved and specialize in presenting to CD1-restricted T cells like double-negative (CD4^−^CD8^−^) and iNKT cells (Arora et al., 2013). CD1a receptors present *Mtb* mycoketides (Moody et al., 2004). CD1d is adapted for presenting phosphoglycolipids such as phosphatidylinositol mannosidase (PIM) (Fischer et al., 2004). Other CD molecules also participate in host cell responses to *Mtb*, with CD1c presenting lipids such as phosphodolichols, phosphomycoketides, and N-terminally acylated lipopeptides (Van Rhijn et al., 2009) and CD1b presenting mycobacterial mycolates and glycolipids, glucose-6-*O*-monomycolate, glycerol monomycolate, and sulfoglycolipids (Moody et al., 1997; Lopez et al., 2020). Invariant NKT cells’ recognition of mycobacterial fractions targets glycolipids *via* CD1d-restricted invariant TCRα chain paired with limited TCRβ chain; the percentage of these cells decreases in active TB as they express programmed death 1 (PD-1) molecule that marks their exhaustion. They are also present in local pleural effusion, where they can produce IL-21, taking part in the stimulation of humoral responses (Wu et al., 2015). Other subtypes of NKT cells produce cytokines like IFN-γ, IL-4, or IL-17a. iNKT cells can directly inhibit the intracellular growth of *Mtb* through the granulocyte-macrophage-colony stimulating factor (GM-CSF) (Rothchild et al., 2014; Di Carlo et al., 2022). Cytokines IL-12 and IL-18 stimulate GM-CSF production by iNKTs. GM-CSF is a potent cytokine involved in macrophage differentiation to M1 phenotype, upregulation of CD11c and MHC II markers and, intracellularly, shifting the balance from antioxidant responses to inflammasome processing and secretion IL-1β while protecting from DNA damages (Di Carlo et al., 2022; Vico et al., 2022). The role of IL-1β in responses to *Mtb* is complex: first in resistance to infection, then protection against cell death, and finally influencing the aforementioned death modality with the shift from necrosis to pyroptosis and the subsequent effect on antigen cross-presentation. Autophagosomal *Mtb* appears to actively counteract host cell inflammasomes *via* modulating its own antigen secretion and interference with host signaling pathways (Rastogi and Briken, 2022). Cytosolic virulent bacilli do the opposite (Beckwith et al., 2020).

Mucosa-associated invariant T cells (MAIT) are present in both the upper and lower respiratory tract and are likely to detect *Mtb* not only in macrophage host but also in infected epithelia while in transit (Harriff et al., 2014). MAIT cells express TCR receptors of restricted diversity and recognize small-molecule microbial metabolites (Chen et al., 2017) presented on MR1 receptors. MR1 ligands in *Mtb* infection are derivatives of riboflavin and folic acid synthesis pathway and possibly other transiently expressed molecules of mycobacterial metabolome like photolumazine I (Harriff et al., 2018). *Mtb* genome contains a family of RibA-H genes, indicating an essential requirement for riboflavin endogenous biosynthesis by the pathogen in the absence of a riboflavin transporter to acquire it or transport it back to the host (Long et al., 2010). The utilization of riboflavin derivatives intersects with iron and bacterial coenzyme F_420_ redox metabolism. Microbial colonization of mucosal surfaces drives the expansion of MAIT cells; there is a proportionally higher abundance of MAIT cells in the jejunum than in lung tissue (Provine and Klenerman, 2020), and overexpression of MR1 ligands has been shown to provide higher protection against TB disease in preclinical models (Dey et al., 2022). The phenotype of MAIT cells is defined as CD161hiVa7.2+ T cells, predominantly CD8^+^. These cells, abundant in the blood, periphery (Greene et al., 2017), and nonlymphoid organs, respond locally and at an earlier stage than the adaptive CD8^+^ ones (Godfrey et al., 2019; Provine and Klenerman, 2020). MAIT cells, similar to classical CD8^+^ cells, produce both cytotoxic granules and cytokines—TNF-α, IFN-γ, IL-17A, IL-2, IL-22, and IL-13, depending on the local microenvironment stimuli, but unlike MHC-Ia-restricted CD8^+^ cells, they are capable of effector functions immediately after leaving the thymus. They have a primarily effector memory phenotype, contribute directly to mycobacterial burden reduction (Chua et al., 2012), and are involved in inflammatory responses to infection.

γδ T cells are a subset of lymphocytes that enrich epithelial tissues. Their major blood subset of Vγ9Vδ2 cells can directly recognize microbial phosphoantigens in a non-MHC-dependent fashion; Vγ9Vδ2 T cells recognize not only molecules belonging to the family of butyrophilin but also mycobacterial 6-*O*-methyl-glucose containing lipopolysaccharides and phosphomonoester molecules. These are known as phosphoantigens, byproducts of the *Mtb* mevalonate metabolic pathway. The smaller periphery-bound subset of Vδ1 T cells bind to antigens displayed by several subtypes of MHC-I-like molecules, i.e., MHC-I chain-related gene A (MICA), *via* the NKG2D receptor shared also with CD8^+^ and NK cells, CD1, and endothelial protein C receptor (EPCR) (Witherden and Havran, 2012). Like other potentially highly cytotoxic cells, γδ T cells express CD94/NKG2A that inhibits MHC-I cells’ destruction by Vγ9Vδ2 ones. All γδ T cells are endowed with innate immune functions, allowing them to directly lyse infected cells as well as produce cytokines that stimulate αβ T cells.

Although not the subject of this review, it is worth remarking that CD4^+^ T cells can also display cytotoxic properties, and the plasticity of the immune response can include smaller populations of either double-negative T cells or MHC-II-restricted CD8^+^ T cells. Linked *via* their cytotoxicity properties are also NK cells, which contribute to overall immune responses to *Mtb*. Leader signal peptides presented *via* HLA-E downregulate NK cells *via* CD94/NKG2A receptors when processed *via* TAP antigen processing machinery. In cases of TAP inhibition, HLA-E is more likely to present exogenous antigens.

## Therapeutic trio: MHC-I antigen processing for TB vaccine development

Despite considerable efforts in constructing a vaccine prototype that would effectively stimulate cytotoxic T-cell responses, the positive results have been moderate so far (Behar et al., 2007) and usually more pronounced in preclinical testing than as an outcome in immunogenicity testing of clinical trial samples (Rodo et al., 2019). Although it gets more accepted that protective immunity to TB may include other than CD4^+^ cell subsets, aggregated CD8^+^ responses constitute, so far, the main readout for current TB vaccine candidates, as summarized in **Table 2**.

**Table 2:** Summary of selected clinical trials for new vaccines against TB that include outcomes related to the cytotoxic T cell.

Vaccine	Strategy tested in a clinical trial		Reference
Viral vector	Trial population	Outcomes related to CTL detection	
**MVA85A** Modified Vaccinia Ankara virus (MVA): attenuated, replication-deficient poxvirus expressing Ag85A	1. Phase I (NCT00460590): healthy adolescents/adults, any BCG status Phase I (NCT00480558): asymptomatic adults: any BCG status: LTBI, HIV+ only, LTBI+HIV+ 2. Phase I/II (NCT03681860): safety and immunogenicity of: MVA85A/ChAdOx1 85A/BCG re-vaccination in healthy, BCG-vaccinated adolescents and adults 3. Phase I trial (NCT01829490) in healthy BCG-vaccinated (up to 6 months before the study) adults; ChAdOx1 85A/ChAdOx1 85A+ MVA85A (boost)	1. Low level of CD8^+^ IFN-γ^+^ and TNF-α^+^ 2. Induction of Ag85A-specific polyfunctional IFN-γ^+^, TNFα^+^ CD8^+^ T cells boosted by MVA85A 3. Ag85A-specific CD8^+^ polyfunctional T cells highest with regimen ChAdOx185A + MVA85A	McShane et al. (2005) and Ndiaye et al. (2015)
**ChAdOx185A** Recombinant vector formed of simian adenovirus and MVA expressing Ag85A			Dicks et al. (2012); Stylianou et al. (2015), and Wilkie et al. (2020)
**Ad5 Ag85A** Recombinant human type 5 adenovirus-expressing Ag85A	Phase I trial (NCT00800670): healthy adults with any BCG status	CD8^+^ was detected in BCG+ individuals with a peak at 2 weeks and sustained TNF-α^+^ IL-2^+^ secretion	Smaill et al. (2013)
**TB-FLU-04L** A negative, single-stranded RNA virus attenuated and genetically manipulated to express TB genes	Phase I study (NCT02501421): BCG vaccinated (up to 6 months before the study) healthy adults	Reported CD4^+^/CD8^+^ antigen-specific responses with a peak at 21 days	Walker et al. (2016)
Recombinant BCG	1. Phase I trial (NCT00749034): healthy adults (Germany), any historic BCG but not in the last 10 years 2. Phase Ib trial (NCT01113281): healthy adults (South Africa), any historic BCG but not in the last 10 years 3. Phase II trial (NCT01479972): newborn infants, BCG naïve (South Africa)	1. Reported increase in proliferative CD8^+^ responses at days 57 and 180 with an increase in multifunctional CD8^+^ at days 29 and 57 2. No reported significant CD8^+^ responses 3. Reported increase in CD8^+^IL-17^+^ at 16-week and 6-month timepoint but not CD8^+^ IFN-γ, TNF-α, or IL-2 either single or multifunctional	Grode et al. (2005); Farinacci et al. (2012); Grode et al. (2013); Saiga et al. (2015); Hoft et al. (2016); Loxton et al. (2017), and Nieuwenhuizen et al. (2017)
**VPM1002** Recombinant BCG with urease C-deficient listeriolysin O			
Subunit antigen and adjuvant	1. Phase IIa trial (NCT00600782) in healthy adults, any BCG status 2. Phase II trials (NCT00621322) in healthy adults, any BCG status 3. Phase IIb trial (NCT01755598; healthy adults, any BCG status with add-on sub-study for biomarkers NCT02097095	1. Reported monofunctional CD8^+^, IFN-γ^+^, TNF-α^+^, IL-2^+^, and IL-17^+^, 7 days after each vaccine dose with pattern indicated boosting of pre-existing responses rather than the generation of *de novo* ones; no effect on PD-1 upregulation; CD8^+^Ki67^+^ upregulation in TST > 10mm group 2. No reported significant CD8^+^ responses 3. No reported significant CD8^+^ responses	Didierlaurent et al. (2017)
**M72-AS01:** Antigens PPE18 and PepA with the liposome-based adjuvant AS01: monophosphoryl lipid A and saponin QS21			
**ID93+GLA-SE** **GLA-SE:** TLR4 agonist glucopyranosyl lipid/CpG ODN. **ID93:** PPE42, esxV, esxW, Rv1813	1. Phase Ib trial (NCT01927159) in healthy, BCG-vaccinated adults (not in the last 5 years). 2. Phase I trial (NCT01599897) in healthy adults; BCG naïve	1. Reported very low and not statistically different IFN-γ, TNF-α, IL-2, and IL-17 CD8^+^ 2. Baseline to very low CD8^+^ responses detected	Coler et al. (2018); Penn-Nicholson et al. (2018), and Kwon et al. (2019)

### Antigen

*Mycobacterium tuberculosis* consists of over 4,000 open reading frames (ORF). Currently, there are 11 antigens in the clinical testing phase. Broadly summarizing, the selection of new candidates includes MHC-I-directed methods and T-cell-specific methods. The former includes bioinformatics approaches of antigen selection based on the prediction databases as one category and immunopurification of naturally presented epitopes in infected cell lines as another. T-cell-specific methods use T cells from infected patients to evaluate their proliferation and polyfunctionality when exposed to peptide pools of selected antigens. The aim is to find antigens that sensitized immune cells respond to the most (Tang et al., 2011; Lewinsohn et al., 2013). In view of conserved immunodominant epitopes present in many of the *Mtb* virulent proteins that participate at early stages of the infection, isolation of antigen-specific T cells is likely to result in the identification of these highly virulent secreted proteins. Indeed, these are the antigens that have been so far tested in clinical trials, with TB10.4 being one of them. Although some of them are still in testing, antigens abundant in subdominant epitopes that elicit weaker natural immune responses are currently considered to be better vaccine candidates (Orr et al., 2014). That reverses the hierarchy of importance between results of *in vitro* T-cell stimulation assays and the validation of antigens as new vaccine candidates. More recently, CD8^+^T- cell-specific methods (Lewinsohn et al., 2017) identified members of the PE-PPE family currently tested as potential vaccine candidates (Stylianou et al., 2018). Improvements in analytical technology, mainly mass spectrometry (Purcell et al., 2019), led to reinvigorated research in immunoproteomics and related immunopeptidomics. In the tuberculosis field, the first demonstrations to identify *Mtb*-specific antigens showed not only many secreted *Esx* family of protein members but also membrane-associated proteins and some molecules involved in lipid biosynthesis and transport. That confirmed the potential usefulness of this method, promising new avenues for further research (Bettencourt et al., 2020).

### Vaccine delivery system and adjuvants

The methods used to boost MHC-I antigen presentation and CTL responses include viral vectors for delivery of mycobacterial antigens (Stylianou et al., 2015; Humphreys and Sebastian, 2018) and adjuvants like the AS01 system deployed in the M72-AS01vaccine, as shown in **Table 2**. AS01 adjuvant consists of saponin extract from *Quillaja saponaria*, QS21, and was reported to strongly induce CD8^+^ responses *via* antigen cross-presentation (Garçon and Van Mechelen, 2011). On the other hand, the *Listeria monocytogenes* toxin, listeriolysin O, which acts as a membrane hole puncher to release live bacilli into the cytosol, was also deployed in combination with BCG to improve cytosolic processing and antigen presentation in the VPM1002 vaccine (Farinacci et al., 2012; Saiga et al., 2015; Nguyen et al., 2019).

### Nonclassically restricted cytotoxic T cells in TB vaccine responses

Nonclassically restricted cytotoxic cells are potent effector cells endowed with variable levels of memory-like functions. Their residency in the periphery and in blood, cytotoxic properties, and lack of donor MHC-I allelic restriction make them potentially first responders to infection and therefore an attractive target for vaccine strategies. As endowed with highly cytotoxic properties and abundantly present at the periphery, they are also tightly controlled in the environment of multimicrobial presence and sensitive tissue to protect mucosal integrity as a mechanical barrier.

Early attempts at the deployment of MR1 ligands in TB vaccine research in the macaque model of TB led to MAIT cell dysfunction and indicated a narrow margin between lung MAIT cell stimulation and exhaustion in this model (Sakai et al., 2021). Lack of margin could be potentially disadvantageous from the therapeutical point of view, rendering MAIT cells easily overstimulated. MAIT cells were also functionally impaired and displayed exhaustion markers in HIV/SIV-*MTB* co-infection in the cynomolgus macaque model, although SIV did not prevent MAIT recruitment from blood to sites of infection in the lungs (Ellis et al., 2020).

In humans, proinflammatory, cytokine-secreting TRAV1-2+ CD8^+^ CD26^+^MAIT cells were identified in the lungs of individuals with active TB (Wong et al., 2019) while depleted in the blood. Transcriptomic analysis of blood samples from LTBI versus noninfected individuals showed a lower frequency of MR1tet+ CD8^+^ cells in LTBI (Pomaznoy et al., 2020), but in another study, the correlation between blood MAIT frequency and TB status was completely absent for both active and latent TB individuals (Suliman et al., 2020). The frequency of MAIT cells was unchanged in samples from the phase I study [NCT01119521] investigating the safety and reactogenicity of BCG revaccination with isoniazid pretreatment in LTBI adults, although changes were observed in the usage of TCR clonotypes (James et al., 2022), indicating the changes in MAIT cells are discrete and qualitative rather than quantitative.

The first identification of mycobacterial antigens stimulating γδ T cells showed the abundance of γδ T cells is proportional to mycobacterial pathogenicity (Constant et al., 1995). It was also observed that this subset of T cells is inducible by cross-reactive antigens from environmental mycobacteria, which at least partially explains the confounding results in the studies measuring their level as a correlate of vaccine efficacy (Hoft et al., 1998). γδ T cells from BCG-vaccinated responders show reactivity to whole-cell *Mycobacterium tuberculosis* lysates rather than secreted components of the culture filtrate or heat-inactivated whole bacilli. They display memory-like phenotype and support the expansion of CD4^+^ and CD8^+^ cells by secretion of IFN-γ. Partially due to the cross-reactivity in small metabolite molecules’ metabolism, BCG-specific γδ T cells are currently investigated as nonspecific immunomodulators to high-grade nonmuscle invasive bladder cancer and HIV-infected cells (Garrido et al., 2018).

NKT-like cells, defined as CD3^+^ and CD56^+^ expressing IFN-γ, TNF, and IL-2, were increased after vaccination with H4:IC31. H4 consists of Ag85B and TB10.4 and H4:IC31, a prototype of H56:IC31, was dropped off the WHO TB new vaccine pipeline in 2018 (WHO, 2017). The H4:IC31 phase I trial [NCT02075203] was composed of the interventional arm testing the H4:IC31 vaccine, while the comparator arms for this study were placebo and BCG revaccination (1:1:1). The immunogenicity outcomes included CD4^+^ and CD8^+^ as main outcome readouts, and a further in-depth flow cytometry strategy was designed to detail all antigen-specific responders. Indeed, the analysis showed that BCG revaccination stimulated donor-unrestricted responses at just slightly lower levels than conventional CD4^+^ T cells. It showed equal proportions of the presence of γδT cells and MAIT cells alongside the same level of innate NK cells. These cells were predominantly monofunctional IFN-γ producers (Rozot et al., 2020).

Finally, linked by their cytotoxic properties rather than lineage, NK cells appear overlooked yet potentially important strategic partners for new vaccine candidates. CD27^+^NK cells accumulated in the LTBI model of nonhuman primates (Esaulova et al., 2021), as well as being present in patients with active TB, where they appeared to enhance the cytotoxicity of CD8^+^ T cells with potential for innate-like memory (Choreño Parra et al., 2017). BCG revaccination increased the number of IFN-γ-producing NK cells and was linked to the nonspecific expansion of this subset in the H4:IC31 vaccine administration (Rozot et al., 2020).

## Summary and conclusions

*Mycobacterium tuberculosis’* adaptation to its host has been refined through thousands of years of coevolution. The renewed interest in intracellular antigen processing and presentation on MHC-I molecules has arisen in an attempt to better define immune correlates of protection against TB infection. Although classical CD8^+^ cells are still considered the main effector for peptides presented by MHC-I molecules and one of the main outcomes for immunogenicity assays, growing interest in donor unrestricted T cells (DURTs) may soon change that readout (Gela et al., 2022). In the majority of already completed trials, BCG vaccination is either a comparator or an inclusion criterion for patients’ eligibility. It is also a frequent *Mtb* surrogate for any *in vitro* and preclinical experiments. Although BCG-induced immune responses set a high testing threshold of efficacy for any new vaccine candidate entering clinical testing, our incomplete understanding of how these immune responses are induced and why they are insufficient in many TB-endemic countries outlines questions yet to be answered both scientifically and therapeutically. Together with new technological developments in cytometry, forthcoming clinical trials indicate a dynamic landscape in TB vaccinology with new, yet unexplored directions ahead.

## Author contributions

The author confirms being the sole contributor of this work and has approved it for publication.
